# Safety of zidovudine/lamivudine scored tablets in children with HIV infection in Europe and Thailand

**DOI:** 10.1007/s00228-016-2182-2

**Published:** 2016-12-27

**Authors:** The European Pregnancy and Paediatric HIV Cohort Collaboration (EPPICC) study group in EuroCoord, Heather Bailey, Lindsay Thompson, Tristan Childs, Intira Jeannie Collins, Anna Tostevin, Ruth Goodall, Tessa Goetghebuer, Vana Spoulou, Luisa Galli, Magda Marczynska, Laura Marques, Luminita Ene, Anna Samarina, Vladimir Rosenberg, Konstantin Dodonov, Liubov Okhonskaia, Antoni Noguera Julian, Pablo Rojo Conejo, Jose Tomas Ramos Amador, Lars Naver, Gonzague Jourdain, Claire Thorne, Carlo Giaquinto, Ali Judd

**Affiliations:** MRC Clinical Trials Unit at UCL, Institute of Clinical Trials and Methodology, Ali Judd, University College London, Aviation House, 125 Kingsway, London, WC2B 6NH UK

**Keywords:** Pharmacovigilance, Children, Antiretroviral therapy, NRTIs, HIV

## Abstract

**Background:**

Zidovudine (ZDV) has been associated with risk of haematological toxicity. Safety data from clinical trials is generally limited to 48 weeks. We assessed the short- and mid-term toxicity of ZDV/lamivudine (3TC) fixed-dose combination scored tablets in HIV-infected children followed in the European Pregnancy and Paediatric HIV Cohort Collaboration (EPPICC) network.

**Methods:**

Fourteen cohorts provided data on patients <18 years of age taking ZDV/3TC scored tablets between 2008 and 2012. Rates of Division of AIDS (DAIDS) grade ≥3 laboratory adverse events (AEs) for hepatobiliary and haematological disorders were estimated by duration on drug (<12, 12–24, >24 months). Clinical adverse events and reasons for tablet discontinuation were described.

**Results:**

Of 541 patients on ZDV/3TC, 388 (72%) had weight and dose data available, of whom 350 (90%) weighed ≥14 kg and were eligible for tablet use; 161 (41%) were aged <10 years on an approved dose, 189 (49%) aged ≥10 years on an approved dose, and 30 (8%) were on an unapproved dose. Median age at ZDV/3TC start was 10 years, and 79% had taken ART previously (60% had prior exposure to ZDV/3TC). Overall rates of grade ≥3 AEs for absolute neutrophil counts, bilirubin, haemoglobin, platelet counts, white blood cell counts (WBC), alanine aminotransferase (ALT) and aspartate aminotransferase (AST) were ≤2/100 person years (PY) for patients taking approved doses. Two hundred thirty-three (43%) patients were not on ZDV/3TC tablets at most recent follow-up; a small number (17 (7%)) discontinued due to AEs (17 (7%)), and the most common reason for discontinuation was treatment simplification (73 (31%)).

**Conclusions:**

Scored ZDV/3TC tablets, both approved and taken off-label, appear to be well tolerated with few side effects. Few patients discontinued treatment due to toxicity. As ZDV/3TC tablets are taken with other antiretrovirals, it is difficult to infer association between toxicities and specific agents, highlighting the importance of widening long-term pharmacovigilance to a broader spectrum of drug combinations.

## Introduction

Zidovudine (ZDV)/lamivudine (3TC) is a first-line NRTI backbone for paediatric HIV infection. A fixed-dose combination of 300 mg ZDV with 150 mg 3TC (Combivir®) was initially approved in Europe in 1998. Subsequently, a scored tablet formulation of ZDV/3TC was approved in 2008 for paediatric HIV treatment for those weighing ≥14 kg or requiring dose adjustment, or patients experiencing dose-limiting adverse reactions. Dosing of ZDV/3TC is by weight bands [1].

ZDV has been associated with the risk of haematological toxicity [2], and WHO guidelines recommend avoiding ZDV for first-line treatment for patients with severe anaemia [1]. However, the ARROW and CHAPAS-3 trial data [3, 4] indicated no increased anaemia risk among children on ZDV, suggesting this may be caused predominantly by chronic HIV or other infections rather than ART. The CNAA3006 study provided safety data on the use of ZDV/3TC in patients aged 6 months to 13 years; however, this study did not evaluate the scored tablet formulation [5]. They found that ZDV/3TC was well tolerated with <10% of participants experiencing a treatment-limiting adverse event (AE) and only 3% of patients experiencing a grade 3/4 anaemia abnormality.

Longer-term follow-up is essential to understand the safety of antiretroviral drugs beyond the 48 weeks of licencing trials. We investigated the short- and mid-term toxicity among children receiving ZDV/3TC scored tablets as part of routine care. Patients were followed in observational cohorts participating in the European Pregnancy and Paediatric HIV Cohort Collaboration (EPPICC), within which a pharmacovigilance programme has been running since 2009 [6, 7].

## Methods

HIV-infected paediatric patients, aged <18 years from 14 cohorts participating in EPPICC’s pharmacovigilance programme (part of the EuroCoord network) who ever received ZDV/3TC scored tablets between 2008 and 2012, were included. Individual participating cohorts were responsible for gaining their own ethics approval. Data collected included demographics, deaths, losses to follow-up and follow-up data (both pre- and post-ZDV/3TC scored tablets) for patient weight, ART, Centres for Disease Control and Prevention (CDC) category C (AIDS) events, CD4 and HIV-1 RNA, key haematology and biochemistry results and serious adverse events (SAEs). Data up to February 2014 were merged, using the HIV Cohorts Data Exchange Protocol specification (www.hicdep.org).

Patients were considered to be on an approved dose if taking ZDV/3TC scored tablets as licenced, i.e. ≥14–< 20 kg, half a tablet (75/150 mg) b.i.d; ≥20–< 30 kg, half a tablet (75/150 mg) in morning, whole tablet (150/300 mg) in evening; and ≥30 kg, whole tablet (150/300 mg) b.i.d. Patients on other doses within these weight bands or weighing <14 kg were considered to be on an unapproved dose.

Biochemical results were graded using the Division of AIDS (DAIDS) categorisation for paediatric AEs (Appendix Table 2) [8]. Rates of grade ≥3 AEs were calculated within four distinct time periods on scored tablet (<12, 12–24 and >24 months) and age starting scored tablet (<10 and ≥10 years) where there were *n* ≥ 30 patients in each group in follow-up for approved doses. Laboratory analyses were restricted to patients with ≥3-month follow-up after start of the tablets and were censored at 30 days after tablet discontinuation. Patients were censored at their first grade ≥3 event within each time period. Clinical SAEs and reasons for tablet discontinuation were also described. Analyses were undertaken using Stata version 14.0 (Stat Corp, College Station, TX, USA).

## Results

Overall, 541/3139 (17%) patients <18 years on ART in 14 cohorts ever took ZDV/3TC scored tablets between 2008 and 2012. The majority were from the Thai (38%), UK/Ireland (18%) and Russian (17%) cohorts, and among those with known mode of HIV acquisition, almost all were perinatally HIV-infected (Table 1). Twenty-five (5%) patients were co-infected with hepatitis-C virus and 23 (4%) with hepatitis-B virus. Of the 145 (27%) patients ever diagnosed with AIDS, 22 (15%) were after tablet start. At the start of ZDV/3TC scored tablets, 190 (35%) had previously received 1–3, 215 (40%) 4–7, and 24 (4%) ≥8 ART drugs; 327 (60%) ZDV and 3TC.

**Table 1:** Characteristics of patients taking ZDV/3TC scored tablets (*n* = 541)

	*N* (%) / median [IQR]	
Country		
UK/Ireland	96	(18)
Other European countries^a^	152	(28)
Russia	90	(17)
Thailand	203	(38)
Male gender	250	(46)
Ethnic group		
White	151	(28)
Black African	104	(19)
Asian and other	236	(44)
Unknown	50	(9)
Mode of HIV infection		
MTCT	450	(83)
Other	13	(2)
Unknown	78	(14)
Ever AIDS event	145	(27)
Median age at ART start (years)	6	[2, 9]
Median age at ZDV/3TC scored tablet start (years)	10	[7, 13]
ART experienced (≥1 ART drug) before ZDV/3TC scored tablets	429	(79)
Median duration on ART before ZDV/3TC scored tablet start (years)	4	[1, 6]
Exposure to ZDV and 3TC before starting ZDV/3TC scored tablets	327	(60)
Median VL at ZDV/3TC start (log_10_c/mL)	1.7	[1.6, 3.3]
Median CD4 cell count ZDV/3TC start (cells/mm^3^)	660	[416, 972]
Median CD4% at ZDV/3TC start	28	[19, 34]
Median time on ZDV/3TC scored tablets (months)^b^	30	[17, 56]
Time to discontinuation of ZDV/3TC (*n* = 233)		
<1 month	11	(5)
1–<6 months	29	(12)
6–<12 months	32	(14)
≥12 months	161	(69)
Reasons for stopping tablets (*n* = 233)		
Treatment failure (immunological/virological)	42	(18)
Toxicity/side effects	17	(7)
Death	7	(3)
Non-compliance	9	(4)
Patient’s wish/decision	7	(3)
Co-morbidity	1	(0)
Physician’s decision	3	(1)
Simplified treatment available	73	(31)
Better safety profile	1	(0)
Unknown	73	(31)

^a^Other European countries are Belgium, Greece, Italy, Poland, Portugal, Romania, Spain and Sweden

^b^For those still on ZDV/3TC at the last follow-up

Overall, 388 (72%) had weight and dose data available at start of ZDV/3TC tablets, of whom 350 (90%) weighed ≥14 kg and were taking an approved dose for weight (161 (46%) aged <10 years and 189 (54%) ≥10 years); 30 (8%) weighed ≥14 kg and were taking an unapproved dose for weight (26 patients were under-dosed and 4 were over-dosed), and 8 (2%) had weight <14 kg on any dose. Thirty-two percent of patients <18 years on ART were taking an NNRTI and 60% a boosted PI.

**Fig. 1:**
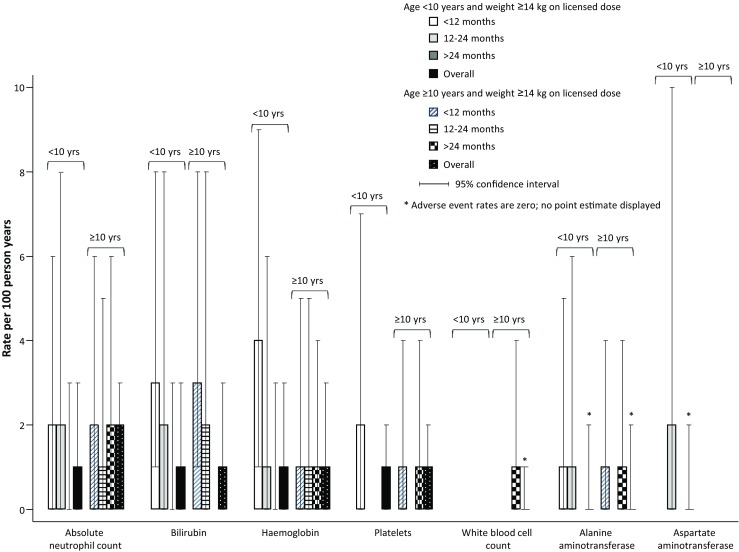
Incidence of grade ≥3 adverse events by duration of ZDV/3TC scored tablets (*n* = 145 < 10 years with weight ≥14 kg, *n* = 166 ≥ 10 years with weight ≥14 kg)

The incidence rates of DAIDS grade ≥3 events were generally low (Fig. 1). Five (4%) patients aged <10 years and 6 (4%) ≥10 years at start of ZDV/3TC tablets had grade ≥3 neutropenia. Two (1%) patients aged <10 years had a grade ≥3 alanine aminotransferase (ALT) event (one <12 and one 12–24 months after starting ZDV/3TC) and two (1%) ≥10 years. One patient aged <10 years (<1%) had a grade ≥3 aspartate aminotransferase (AST) event 12–24 months after starting ZDV/3TC. Five (4%) patients aged <10 years had grade ≥3 hyperbilirubinemia and five patients (4%) ≥10 years (four within 12 months of starting ZDV/3TC). Five (4%) patients aged <10 years had grade ≥3 anaemia (four within 12 months of starting ZDV/3TC) and 4 (2%) ≥10 years. Two (2%) patients aged <10 years had grade ≥3 platelet results (both <12 months after starting ZDV/3TC) and 2 (1%) ≥10 years (1 within 12 months, and 1 after 24 months). There was only 1 grade ≥3 WBC event among patients aged ≥10 years.

Among 22 patients taking unapproved doses of ZDV/3TC with ≥1 laboratory test, 4/15 (20%) had grade ≥3 neutropenia, of which two subsequently resolved; all the four were aged ≥10 years at the time of the grade ≥3 result. One (5%) had grade ≥3 hyperbilirubinemia reported <12 months after starting ZDV/3TC and again at 12–24 months. Of the eight patients with weight <14 kg one had grade ≥3 hyperbilirubinemia.

Among 159 patients on approved dose with clinical data, there were five SAEs causally related to ZDV/3TC, all aged <10 years at start of ZDV/3TC; anaemia (*n* = 4) and neutropenia (*n* = 1). There were no clinical SAEs considered causally related to the tablets for patients aged ≥10 years at start of ZDV/3TC on an approved or unapproved dose.

During follow-up, 12 (2%) patients died, of whom seven were taking ZDV/3TC at death. Nine deaths were patients aged ≥10 years at start of ZDV/3TC and on an approved dose, and the deaths were reported as HIV-related (*n* = 5) or AIDS-defining (*n* = 1) events, suicide (*n* = 1) or unknown causes (*n* = 2; 21 and 22 months after starting ZDV/3TC scored tablets). In the unapproved dose group, two deaths were reported during follow-up, one from HIV-related causes and one an AIDS-defining event*.* One death occurred in the <14-kg weight category and one in a patient with missing dose; one was taking ZDV/3TC at time of death.

 Overall, 233 (43%) patients were not on ZDV/3TC tablets at the last follow-up, and the main reasons for discontinuations were treatment simplification (31%) and treatment failure (18%) (Table 1). Most patients discontinued ≥12 months after starting ZDV/3TC. Seventeen patients discontinued due to toxicity, including abnormal fat redistribution (*n* = 3), dyslipidaemia (*n* = 2) haematological (*n* = 4), liver (*n* = 2), nervous system (*n* = 2), hypersensitivity reaction (*n* = 2) and unspecified (*n* = 2).

## Discussion

The findings from this study suggest that ZDV/3TC scored tablets were received by nearly one in five children currently on ART in cohorts in Europe and Thailand. In patients taking the approved doses, rates of grade ≥3 AEs were generally low and were comparable across children aged <10 and ≥10 years at the start of ZDV/3TC tablets. These results are comparable to those of the CNAA3006 study that showed grade ≥3 AEs occurred in ≤30% of participants and were mild/moderate in intensity; <10% of the participants experienced a treatment-limiting AE. Rates of grade ≥3 AEs were also similarly low to those in children in our collaboration taking fosamprenavir-, darunavir- and atazanavir-containing regimens [6, 7]. Discontinuations in this study, most of which occurred at least 12 months after starting treatment, were mainly due to treatment simplification. A small number of children died, for whom the most common causes were HIV- or AIDS-related. This is comparable with CNAA3006 where there was only 1 death in 103 participants in the ZDV/3TC group, considered unrelated to the study drug.

A limitation of this analysis is that EPPICC cohorts do not routinely capture mild AEs such as diarrhoea, vomiting, nausea and choking; thus, it was not possible to estimate their frequency in this report. Also due to the small number of grade ≥3 lab events, it was not possible to stratify rates by country, or to present rates for those taking unapproved doses. We were also unable to compare characteristics of patients with and without SAEs due to the small numbers. As 60% of patients were exposed to ZDV and 3TC prior to starting ZDV/3TC scored tablets, the incidence of AEs in this population may not be generalizable to children newly exposed to ZDV/3TC. Similarly, since ZDV/3TC is routinely taken with other antiretroviral drugs, it is difficult to attribute causality for these occurrences to any one specific agent, highlighting the importance of widening long-term pharmacovigilance studies to a broader spectrum of drug combinations.

In summary, ZDV/3TC-containing regimens, both approved and unapproved, appear to be well tolerated in the paediatric population in Europe and Thailand. No major post-licencing short- to medium-term safety concerns were identified in our study.

## Appendix

**Table 2:** Division of AIDS table for grading the severity of adult and paediatric adverse events version 1.0, December, 2004; clarification August 2009

Parameter	Grade 1 mild	Grade 2 moderate	Grade 3 severe	Grade 4 potentially life threatening
Absolute neutrophil count				
Adult and paediatric >7 days	1000–1300/mm^3^ 1.000 × 109–1.300 × 109/L	750–999/mm^3^ 0.750 × 109–0.999 × 109/L	500–749/mm^3^ 0.500 × 109–0.749 × 109/L	<500/mm^3^ <0.500 × 109/L
Alanine aminotransferase (ALT)	1.25–2.5 × ULN	2.6–5.0 × ULN	5.1–10.0 × ULN	>10.0 × ULN
Aspartate aminotransferase (AST)	1.25–2.5 × ULN	2.6–5.0 × ULN	5.1–10.0 × ULN	>10.0 × ULN
Bilirubin (total)				
Adult and paediatric >14 days	1.1–1.5 × ULN	1.6–2.5 × ULN	2.6–5.0 × ULN	>5.0 × ULN
Haemoglobin				
Adult and paediatric ≥57 days (HIV positive only)	8.5–10.0 g/dL 5.24–6.23 mmol/L	7.5–8.4 g/dL 4.62–5.23 mmol/L	6.50–7.4 g/dL 4.03–4.61 mmol/L	<6.5 g/dL <4.03 mmol/L
Platelets, decreased	100,000–124,999/mm^3^ 100.000 × 109–124.999 × 109/L	50,000–99,999/mm^3^ 50.000 × 109–99.999 × 109/L	25,000–49,999/mm^3^ 25.000 × 109–49.999 × 109/L	<25,000/mm^3^ <25.000 × 109/L
White blood cells, decreased	2000–2500/mm^3^ 2.000 × 109–2.500 × 109/L	1500–1999/mm^3^ 1.500 × 109–1.999 × 109/L	1000–1499/mm^3^ 1.000 × 109–1.499 × 109/L	<1000/mm^3^ <1.000 × 109/L

(Reproduced from http://rsc.tech-res.com/docs/default-source/safety/table_for_grading_severity_of_adult_pediatric_adverse_events.pdf)
